# Stapled intestinal anastomosis is a simple and reliable method for management of intestinal caliber discrepancy in children

**DOI:** 10.1007/s00383-012-3146-y

**Published:** 2012-08-04

**Authors:** Kaori Sato, Hiroo Uchida, Yujiro Tanaka, Shinya Takazawa, Takahiro Jimbo, Kyoichi Deie

**Affiliations:** Department of Pediatric Surgery, Saitama Children’s Medical Center, 2100 Magome, Iwatsuki, Saitama, Saitama 339-8551 Japan

**Keywords:** Stapled intestinal anastomosis, Infant, Functional end-to-end anastomosis, Caliber discrepancy

## Abstract

**Purpose:**

Popularity of minimally invasive surgeries has led to the development of stapled intestinal anastomosis for adults. The advanced instruments used in this technique are getting suitable with the small intestinal lumens of neonates and infants. We reviewed and compared the intraoperative and postoperative results of stapled and hand-sewn anastomoses in children.

**Methods:**

The operative data of children who underwent stapled and hand-sewn anastomoses between March 2005 and December 2011 were collected and analyzed retrospectively. Furthermore, we compared patients who underwent anastomoses for colostomy closure of anorectal malformation (4 stapled, 9 hand-sewn) and those who underwent anastomoses for treatment of ileal atresia (3 stapled, 11 hand-sewn).

**Results:**

In the 47 patients who underwent stapled anastomosis, no intraoperative complications were observed and postoperative complications included wound infection (*n* = 3), delayed gastric emptying (*n* = 1), and ileus (*n* = 1). No complications suggesting anastomotic dilatation were identified. It was observed that patients who underwent stapled anastomosis for colostomy takedown with caliber discrepancy had significantly shorter surgery time than those who underwent hand-sewn anastomosis.

**Conclusion:**

Our results suggest that stapled anastomosis is safe and effective for various surgical diseases in neonates, infants, and children.

## Introduction

The safety and efficacy of stapled gastrointestinal tract anastomosis in adults have been extensively documented [1]. The recent enthusiasm regarding minimally invasive techniques has led to the development of stapled intestinal anastomosis [2, 3]. The advanced instruments used in this technique may be suitable for the smaller intestinal lumens of neonates and infants. Hand-sewn techniques have traditionally been used to perform intestinal anastomosis in pediatric patients in many cases. When treating small intestinal atresia and stoma closure, great discrepancy between diameters of the proximal and distal intestine caused by disuse atrophy are often observed, which may cause difficulties and complications. To overcome size discrepancy, proficiency in performing anastomosis is required when using hand-sewn techniques [4, 5]. In theory, stapled functional end-to-end anastomosis does not require a special technique and does not impair the passage of intestinal contents immediately after completion because the side-to-side nature of the procedure retains the unique diameter of the target intestine and preserves patency. Stapled side-to-side functional end-to-end intestinal anastomosis is a potentially useful technique that is not affected by intestinal size discrepancy and does not require specialized surgical experience. Stapled anastomosis can be used to perform intestinal anastomosis in children easily and reproducibly. In 2009, we began performing consecutive stapled intestinal anastomosis in newborns, infants, and children. The aim of this study was to assess the feasibility and outcome of stapled intestinal anastomosis in neonates and infants, especially in those who underwent anastomosis for caliber discrepancy, such as for ileostomy/colostomy closure and treatment for small intestinal atresia.

## Patients and methods

All patients who underwent intestinal anastomosis with stapling instruments between April 2009 and December 2011 were retrospectively analyzed, and demographic data, intraoperative results, and outcomes were recorded. Data collected included sex, primary diagnosis, age at surgery, weight, type of anastomosis, surgery time, estimated blood loss, anastomotic leakage, intestinal obstruction, time until initial oral feeding, time of discharge, and postoperative complications.

**Fig. 1:**
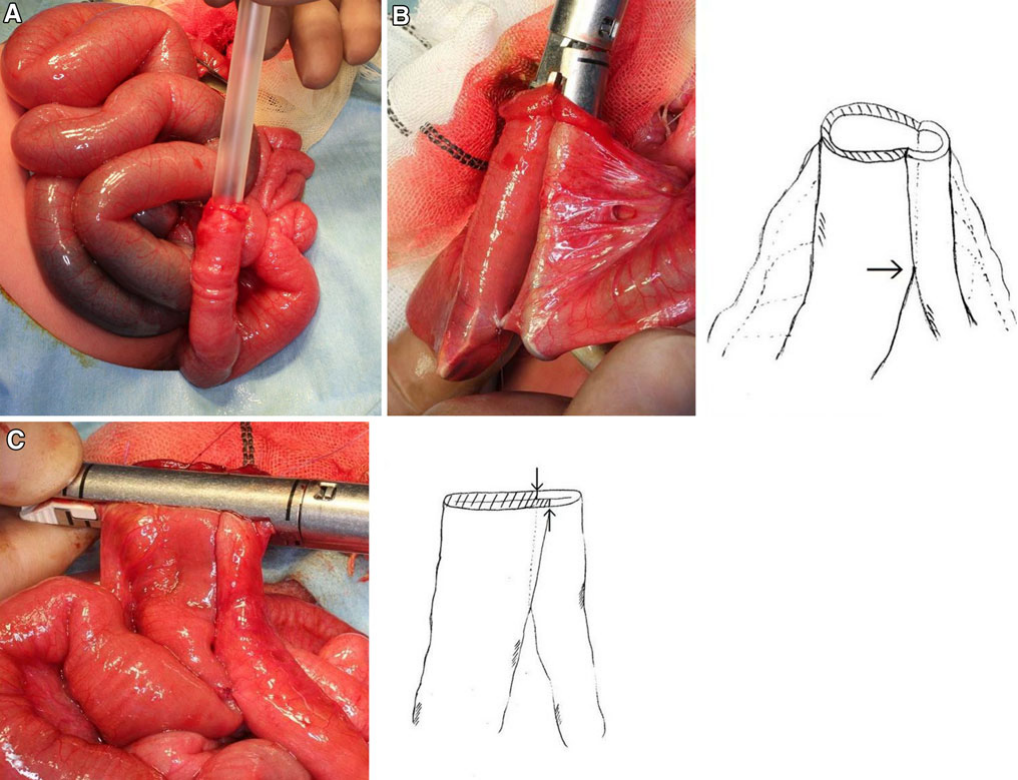
Functional end-to-end anastomosis. **a** 22Fr. soft catheter inserted in the intestinal tract of small diameter. **b** A side-to-side anastomosis is made in both limbs at the anti-mesenteric border. The staple lines are only oversewn at points of bleeding for hemostasis and for reinforcement of the crotch of side-to-side anastomosis (*arrow*). **c** The suture line of side-to-side anastomosis does not overlap when the stapler is fired across the jointed limbs. The staple lines are only oversewn at points of bleeding for hemostasis and for reinforcement of double-stapled areas (*arrows*)

End-to-end and end-to-side stapled intestinal anastomosis methods were used. Functional end-to-end anastomosis was used for intestinal lesions, congenital intestinal atresia, stoma closure after intestinal preparation, and primary bowel resections with subsequent stapled anastomosis. End-to-side anastomosis, also called Roux-en-Y anastomosis, was performed for portoenterostomy, hepatocholangiojejunostomy, and esophagogastric dissociation. An Endocutter ETS 35 or ETS Flex 45 stapler with 1.0 or 1.5-mm staples (Johnson & Johnson K.K., Tokyo, Japan) was used to perform functional end-to-end and Roux-en-Y anastomoses. For very small intestines, an Endocutter stapler was used to perform stapled anastomosis if the intestinal lumens could admit a 22-Fr soft catheter. Stapled anastomosis was contraindicated when the intestinal lumen could not admit a 22-Fr soft catheter or when stapling would significantly compromise the total intestinal length or the ileocecal valve. An important consideration of stapled anastomosis is that the suture line of side-to-side anastomosis does not overlap when the stapler is fired across the jointed limbs, and the staple lines are only oversewn at points of bleeding for hemostasis or for reinforcement of double-stapled areas (Fig. 1).

The subgroups analyzed were patients who underwent intestinal anastomoses in hand-sewn manner, undergoing colostomy closure for anorectal malformation, and undergoing primary anastomosis for congenital ileal atresia from 2005 to 2011. An absorbable suture material was used to perform hand-sewn anastomosis in an end-to-end manner. In general, the method of intestinal anastomosis was based on the surgeon’s preference. Some surgeons did not perform stapled anastomosis, and others always performed stapled anastomosis, when permitted by the intestinal size and surgical circumstances.

We compared patients in the hand-sewn and stapled groups who underwent either colostomy closure for anorectal malformation or treatment for ileal atresia. This comparison was performed to assess the potential benefits of stapled side-to-side functional end-to-end anastomosis for the management of significant size discrepancy between diameters of the proximal and distal intestines. Student’s *t* test and *x* ^2^ test were used to evaluate differences between the groups. *P* < 0.05 was considered to be statistically significant.

## Results

Data of 47 consecutively treated children who underwent procedures requiring stapled intestinal tract anastomosis from April 2009 to December 2011 were analyzed. There were 20 males and 27 females, with age ranging from 0 day to 13 years (mean, 20 months) and weight from 2.8 to 56.2 kg (mean, 10.3 kg). Four (8.5 %) of 47 children were ≤30 days old and had an average weight of 3.1 kg (range, 2.8–3.4 kg). The demographic data and outcomes of functional end-to-end and end-to-side anastomoses are summarized in Table 1. Twenty-five functional end-to-end anastomoses (53.2 %) were performed. Sixteen of 25 cases underwent primary bowel resections and subsequent stapled anastomosis for treatment of ileal atresia (*n* = 3), Meckel’s diverticulum (*n* = 3), intussusception (*n* = 3), malignant lymphoma (*n* = 2), strangulated ileus (*n* = 2), necrotizing enterocolitis (*n* = 1), meconium peritonitis (*n* = 1), and mesenteric cyst (*n* = 1). Nine of 25 patients had intestinal stapling after intestinal preparation during colostomy, ileostomy, or jejunostomy closure. Twenty-two Roux-en-Y anastomoses (46.8 %) were performed during portoenterostomy for biliary atresia (*n* = 9), hepatocholangiojejunostomy for congenital biliary dilatation (*n* = 11), and esophagogastric dissociation for gastroesophageal reflux disease (*n* = 2).

**Table 1:** Patient demographics and outcomes for stapled intestinal anastomosis

	Functional end-to-end anastomosis	End-to-side anastomosis
Number	25	22
Sex	15 (60 %) males	5 (23 %) males
Age at surgery (months)	15.1 ± 20.6	26.7 ± 40.4
Weight at surgery (kg)	8.5 ± 4.7	12.3 ± 12.9
Anastomotic leakage	0	0
Length until initial oral feeding (days)	5.0 ± 2.6	5.8 ± 3.8
Wound infection	3	0
Ileus	1	0
Reoperation	0	0
Mortality	0	0

In majority of the patients, staplers with 1.0-mm staples (*n* = 41) were used to perform stapled intestinal anastomosis. Staplers with 1.5-mm staples were used in the procedures for the remaining six children.

No intraoperative complications were observed in any of the 47 cases. Postoperative complications included wound infection (*n* = 3), delayed gastric emptying (*n* = 1), and ileus (*n* = 1). Every complication was improved by conservative treatment. Until date, we have noted no postoperative intestinal obstruction because of strictures or anastomotic dilatation with subsequent stasis/overgrowth related to anastomosis. The follow-up period ranged from 3 months to 3 years after surgery.

Patients who underwent stapled functional or hand-sewn end-to-end anastomosis with significant size discrepancy were compared. In 13 infants who underwent colostomy closure for anorectal malformation without any other additional surgeries (4 stapled and 9 hand-sewn), no differences in infant size or age was observed between those who underwent stapled and hand-sewn anastomoses; however, the mean surgery time in infants who underwent stapled anastomosis was significantly lower than that in infants who underwent hand-sewn anastomosis. The length of postoperative hospital stay and length until initial oral feeding were shorter in patients who underwent stapled anastomosis, but were not significantly different from those who underwent hand-sewn anastomosis (Table 2). Among the 14 newborns who were treated for ileal atresia (3 stapled and 11 hand-sewn), there were no significant differences in any of the parameters; however, surgery time, length of postoperative hospital stay, and length until initial oral feeding were shorter in those who underwent stapled anastomosis than those who underwent hand-sewn anastomosis. Two patients who underwent hand-sewn anastomoses suffered from anastomotic strictures and delayed oral feeding, but none of the patients who underwent stapled functional end-to-end anastomosis had anastomotic strictures (Table 3).

**Table 2:** Patients undergoing colostomy takedown for anorectal malformation

	Hand-sewn	Stapled	*P*
Number	9	4	
Sex	6 (67 %) males	2 (50 %) males	NS
Age at surgery (months)	10.9 ± 4.8	7.5 ± 2.1	NS
Weight at surgery (kg)	8.0 ± 1.3	8.3 ± 0.6	NS
Surgery time (min)	95.7 ± 14.7	74.8 ± 3.6	0.019
Estimated blood loss (ml)	15.2 ± 10.1	15.7 ± 15.5	NS
Length until initial oral feeding (days)	6.2 ± 3.4	4.0 ± 0.8	NS
Length of postoperative stay (days)	16.1 ± 7.8	11.8 ± 2.4	NS
Anastomotic leakage	0	0	NS
Wound infection	2	1	NS
Ileus	1	0	NS

NS indicates not statistically significant

**Table 3:** Patients undergoing treatment for congenital ileal atresia

	Hand-sewn	Stapled	*P*
Number	11	3	
Sex	4 (36 %) males	0 (0 %) males	NS
Age at surgery (months)	1.9 ± 0.9	0 ± 0	NS
Weight at surgery (kg)	2.8 ± 0.3	3.1 ± 0.3	NS
Surgery time (min)	96.8 ± 3.4	86.0 ± 6.5	NS
Estimated blood loss (ml)	28.6 ± 26.7	19.0 ± 14.9	NS
Length until initial oral feeding (days)	8.5 ± 6.7	3.7 ± 0.6	NS
Length of postoperative stay (days)	21.6 ± 12.0	12.3 ± 4.9	NS
Anastomotic leakage	2	0	NS
Wound infection	0	1	NS
Abdominal abscess	1	0	NS

NS indicates not statistically significant

## Discussion

Stapling devices have a long history in surgery. The safety, efficacy, and technique of stapled gastrointestinal tract anastomosis in adults have been extensively documented since 1978. Several studies, including a Cochrane review of 6 trials that involved 955 ileocolic anastomoses, have suggested that stapled intestinal anastomosis has fewer leaks with no differences in surgery time or the incidence of stricture or wound infection compared with those of hand-sewn anastomosis [6]. On the other hand, hand-sewn techniques have traditionally been used to perform intestinal anastomosis in children. In 1974, Talbert et al. [7] described a modification of the Duhamel procedure that used a linear stapling–dividing instrument. The mechanical stapler for intestinal anastomosis in children had been used in surgeries for a limited number of diseases, such as Hirschsprung’s disease. Few studies have documented stapled anastomosis for other diseases in children. In 1995, Powell published a series of seven successful intestinal anastomoses that used mechanical staplers in infants less than 4 months old [8]. In 2008, Wrighton et al. reported an experience with stapled intestinal anastomosis in infants less than 1 year old, and compared surgical data and outcome with those of infants who underwent hand-sewn anastomosis [9]. In the present study, stapled intestinal anastomosis was shown to have shorter surgery time than hand-sewn anastomosis and similar complications. A recent article by Mitchell et al. [10] documented 64 consecutive stapled intestinal anastomoses. It concluded that stapled anastomosis was an effective approach applicable to various surgical diseases in newborns and infants. In the present study, we found that stapled anastomotic complications were not more severe than those associated with hand-sewn anastomosis.

The recent enthusiasm for minimally invasive surgeries and standardization of surgical procedures may present a need for a simple and reproducible technique of anastomosis in children. In particular, size discrepancy between diameters of the proximal and distal intestines can complicate the surgical anastomotic technique. Several geometrical methods have been used to manage this discrepancy in order to reduce the risk of anastomotic leakage and stenosis [4, 5]; however, there is no ideal technique for the management of size discrepancy. Almost all of the methods should be performed according to the surgeon’s experience and skills. In addition, the nature of hand-sewn techniques makes the site of anastomosis edematous to a greater or lesser extent immediately after completion; however, stapled side-to-side functional end-to-end intestinal anastomosis does not require direct access to the entrance of a disused and distal intestine to maintain patency. The side-to-side stapled anastomosis yields theoretically wider anastomosis than those resulting from the hand-sewn technique. This may account for the lower incidence of strictures found in some adults, although no significant differences were found in obstructive outcomes in our study. We found that surgery time was reduced during colostomy takedown, and anastomotic complications were consistently not more severe than those of the hand-sewn technique. For treatment of congenital ileal atresia, no significant differences were observed in surgery time, length until initial oral feeding, and length of postoperative stay, but our results may indicate the low number of cases. Further studies involving more patients are needed to confirm that surgery time, length until initial oral feeding, and length of postoperative stay are significantly reduced in the stapled group for the patients with caliber discrepancy. Our data may indicate a strong tendency toward good patency for anastomosis with size discrepancy. Functional end-to-end anastomosis may be a superior procedure that anyone can perform simply for intestinal anastomosis with caliber discrepancy compared with hand-sewn anatomosis that are dependent on surgical skills.

Stapled anastomosis could not be performed when the intestinal lumen could not admit a 22-Fr soft catheter. The size limitation of the stapler is a major concern; however, in the review of Wrighon et al. [9], 25 anastomoses were performed in infants between 600 and 1000 g. There may be infrequent cases in which the stapler may not be suitable.

There is only one report of adverse outcomes after stapled intestinal anastomosis in 2-month-old infants and 3-year-old children with partial obstructions after stapled anastomosis [11]. Anastomosis was noted to have dilated significantly, which caused subsequent volvulus. The authors concluded that a functional end-to-end anastomosis may be susceptible to massive dilatation because of the pouch that can be created in the anastomotic region. We must note that functional end-to-end anastomosis should not be excessively long to prevent pouch formation, stasis, and subsequent dilatation.

Our findings showed that in infants who underwent stapled intestinal anastomosis, especially those who underwent colostomy closures with caliber discrepancy, had shorter surgery time than those who underwent hand-sewn anastomosis. There were no differences in adverse outcomes. Stapled functional end-to-end anastomosis does not require a special technique, even if caliber discrepancy is apparent. In conclusion, stapled anastomosis, when permitted by intestinal size, is one of the most simple and reliable methods for intestinal anastomosis, even in newborns and infants with caliber discrepancy. This technique may become an alternative to traditional hand-sewn techniques in small children. In the future, the technique’s impact on long-term outcome should be determined to verify these findings.
